# Supplementary material for: Controlled large non-reciprocal charge transport in an intrinsic magnetic topological insulator MnBi2Te4
Source: Nat Commun. 2022 Oct 19;13:6191. doi: 10.1038/s41467-022-33705-y (PMC9582003; doi:10.1038/s41467-022-33705-y)
Supplement: Supplementary file 1 — Supplementary information [file 41467_2022_33705_MOESM1_ESM.docx]

**Supplementary Information**

**Controlled large non-reciprocal charge transport in an intrinsic magnetic topological insulator MnBi_2_Te_4_**

Zhaowei Zhang^1*^, Naizhou Wang^1*^, Ning Cao^2^, Aifeng Wang^2^, Xiaoyuan Zhou^2^, Kenji Watanabe^3^, Takashi Taniguchi^4^, Binghai Yan^5†^, Wei-bo Gao^1,6,7†^

*^1^ Division of Physics and Applied Physics, School of Physical and Mathematical Sciences, Nanyang Technological University, Singapore 637371, Singapore*

*^2^ Low Temperature Physics Laboratory, College of Physics and Center for Quantum Materials and Devices, Chongqing University, Chongqing 401331, China*

*^3^ Research Center for Functional Materials, National Institute for Materials Science, 1-1 Namiki, Tsukuba 305-0044, Japan*

*^4^ International Center for Materials Nanoarchitectonics, National Institute for Materials Science, 1-1 Namiki, Tsukuba 305-0044, Japan*

*^5^ Department of Condensed Matter Physics, Weizmann Institute of Science, Rehovot 7610001, Israel  ^6^ The Photonics Institute and Centre for Disruptive Photonic Technologies, Nanyang Technological University, Singapore 637371, Singapore*

*^7^ Centre for Quantum Technologies, National University of Singapore, Singapore.*

**These authors contribute equally to this work.*

*†Corresponding author. Email: binghai.yan@weizmann.ac.il, wbgao@ntu.edu.sg*

**Supplementary Note 1: Layer thickness characterisation**

We have fabricated five MnBi_2_Te_4_(MBT) devices, including two 5-SL (Device 1, Device 2, Device 4 and Device 5) and one 4-SL (Device 3) MBT devices. The MBT thin flakes are obtained by Al_2_O_3_ assisted exfoliation method and the thickness of the thin flakes are determined by optical contrast^1,2^. The transmission is defined as *I*_sample_/*I*_substrate_, where *I*_sample_ and *I*_substrate_ are the intensity of the transmission through the sample and substrate respectively, in the green channel of the optical image.

| 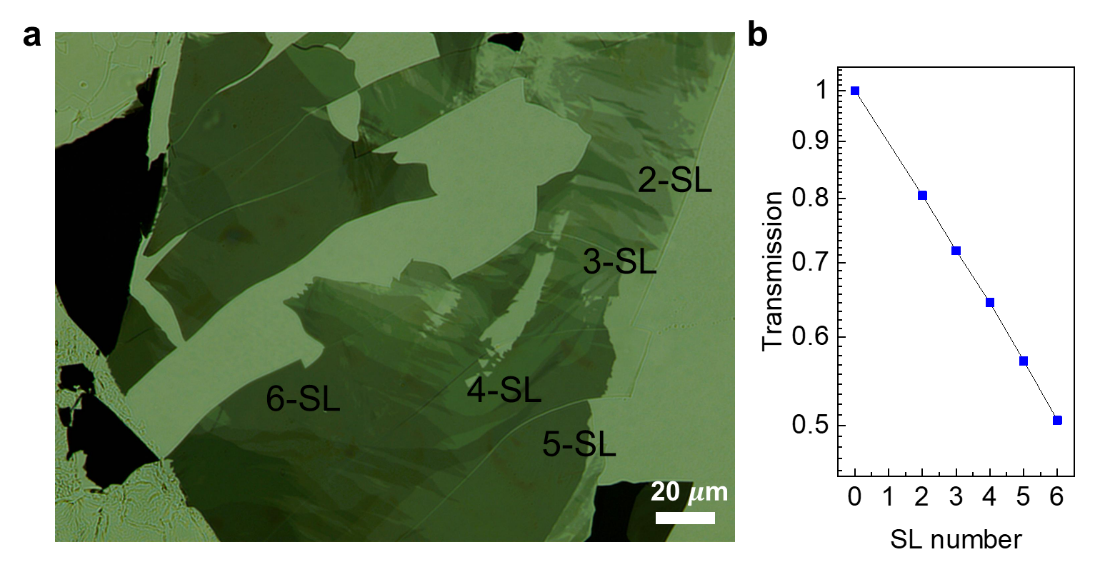 |
| --- |
| **Supplementary Fig. 1: Exfoliation of MBT with Al_2_O_3_ assisted exfoliation method.** **a**, Optical image of the MBT thin flakes on polydimethylsiloxane(PDMS)/Al_2_O_3_ substrates. The layer number is labelled. **b**, Transmission as a function of the number is SLs. |

**Supplementary Note 2: Quantum anomalous Hall(QAH) states in Device 2 and Device 3**

Supplementary Fig. 2 shows the quantum anomalous Hall states of Device 2 and Device 3. Both 5-SL MBT and 4-SL MBT shows the N$\acute{e}$el temperature of 23 K, which is determined by the resistance peak in Supplementary Fig. 2a and d.

We also investigate the effect of gate voltage on the Hall resistance and longitudinal resistance under -7 T magnetic field at 1.7 K. Supplementary Fig. 2a and b show the gate dependence of Device 1 and Device 3. At the charge neutrality point, the Hall resistance reaches the maximum and the longitudinal resistance reaches the minimum.

| 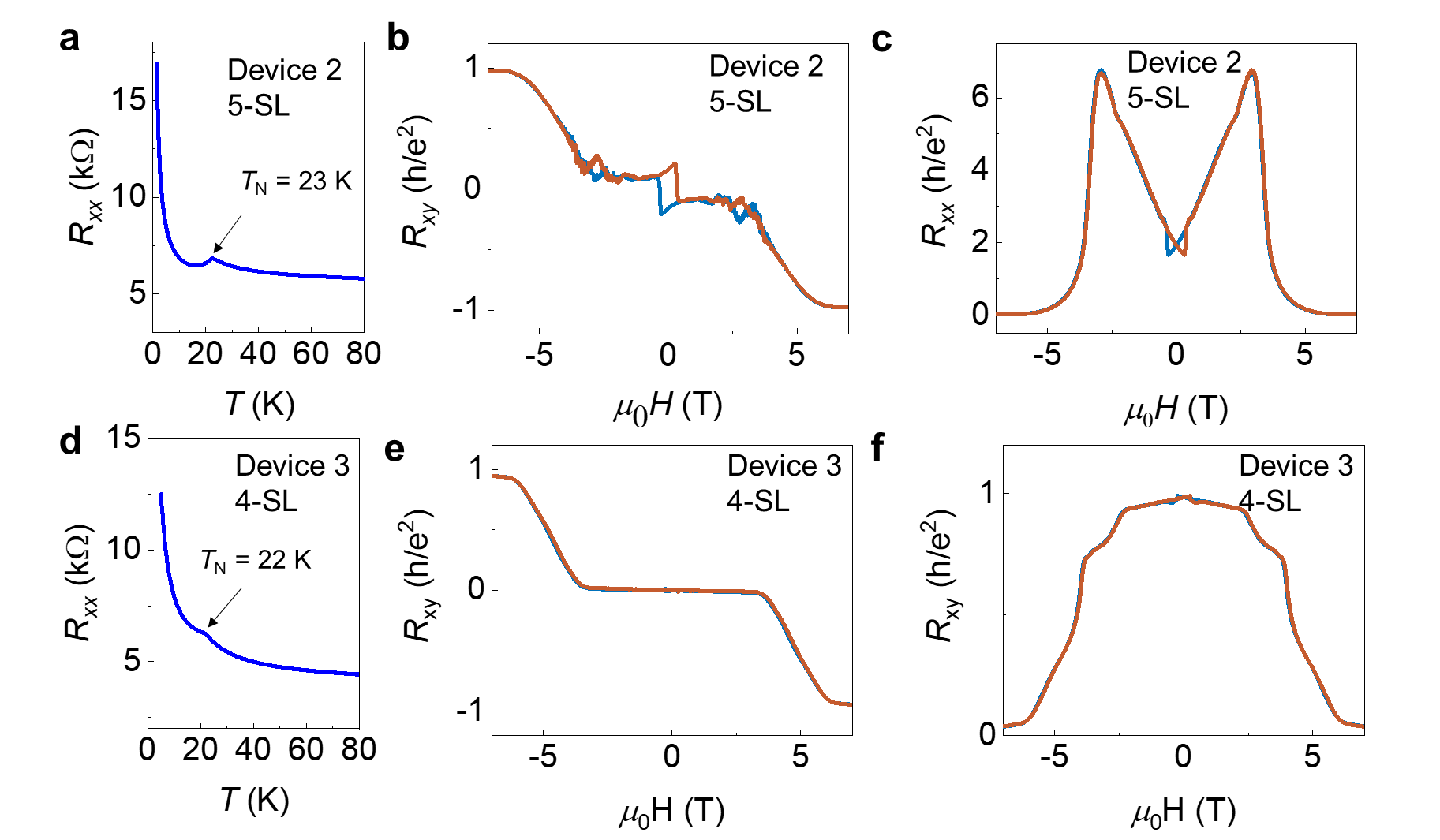 |
| --- |
| **Supplementary Fig. 2: The quantum anomalous Hall states in 5-SL and 4-SL MBT devices.** **a**, **b** and **c** show the temperature dependent resistance, field dependent Hall resistance and longitudinal resistance of Device 2 (5-SL). **d**, **e,** and **f** show the temperature dependent resistance, field dependent Hall resistance and longitudinal resistance of Device 3 (4-SL) |

| 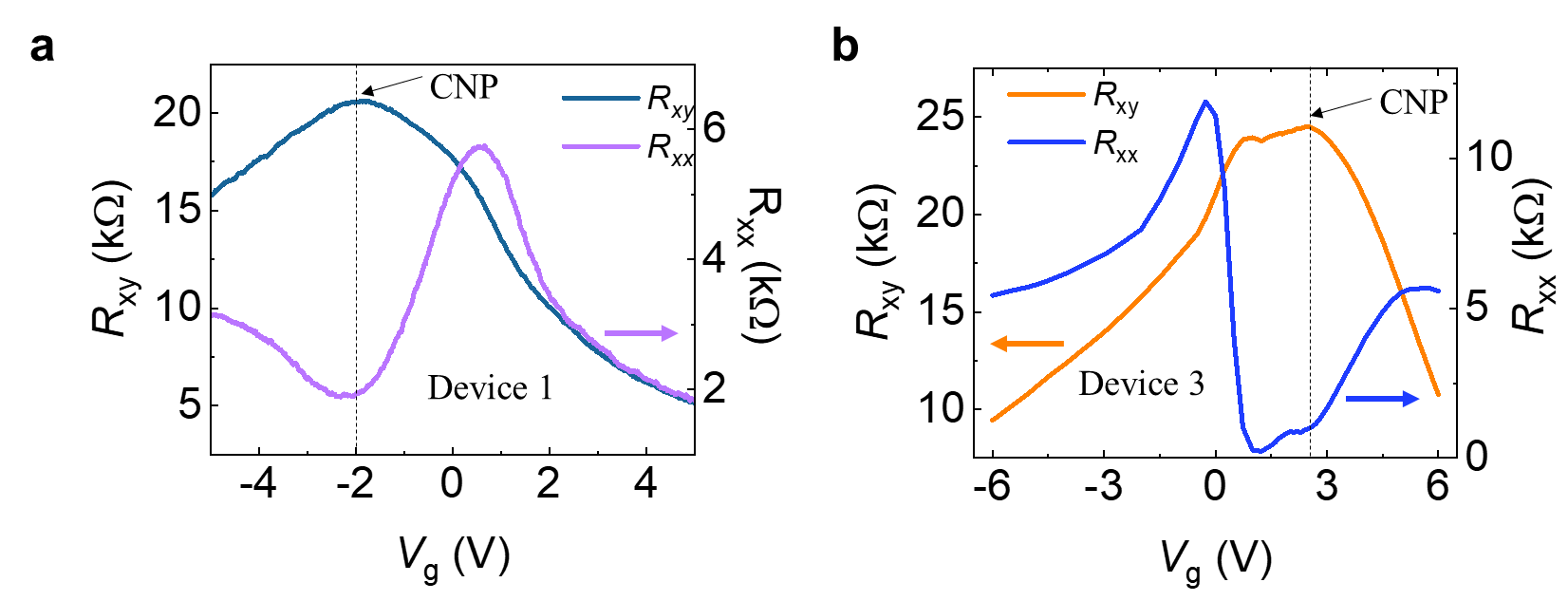 |
| --- |
| **Supplementary Fig. 3: The gate dependence of 5-SL and 4-SL MBT devices.** **a** The gate voltage dependent Hall and longitudinal resistance of Device 1 under -7 T magnetic field at 1.7 K. **b** The gate voltage dependent Hall and longitudinal resistance of Device 3 under -7 T magnetic field at 1.7 K. |

The temperature dependence of Device 1 and Device 3 are shown in Supplementary Fig. 4. Under -7 T out-of-plane magnetic field, increasing the temperature leads to the reduced Hall resistance and the longitudinal resistance is thermally activated following the relation, $R_{xx}\propto e^{\frac{-\Delta}{2k_{B}}}$, where $\Delta$ is the activation energy gap and $k_{B}$ is the Boltzmann constant. By linearly fitting the Arrhenius plot of ln($R_{xx}$) as a function of $\frac{1}{T}$, we estimated the $\Delta$ under -7 T magnetic field to be 0.944 meV in 5-SL and 0.779 meV in 4-SL MBT.

| 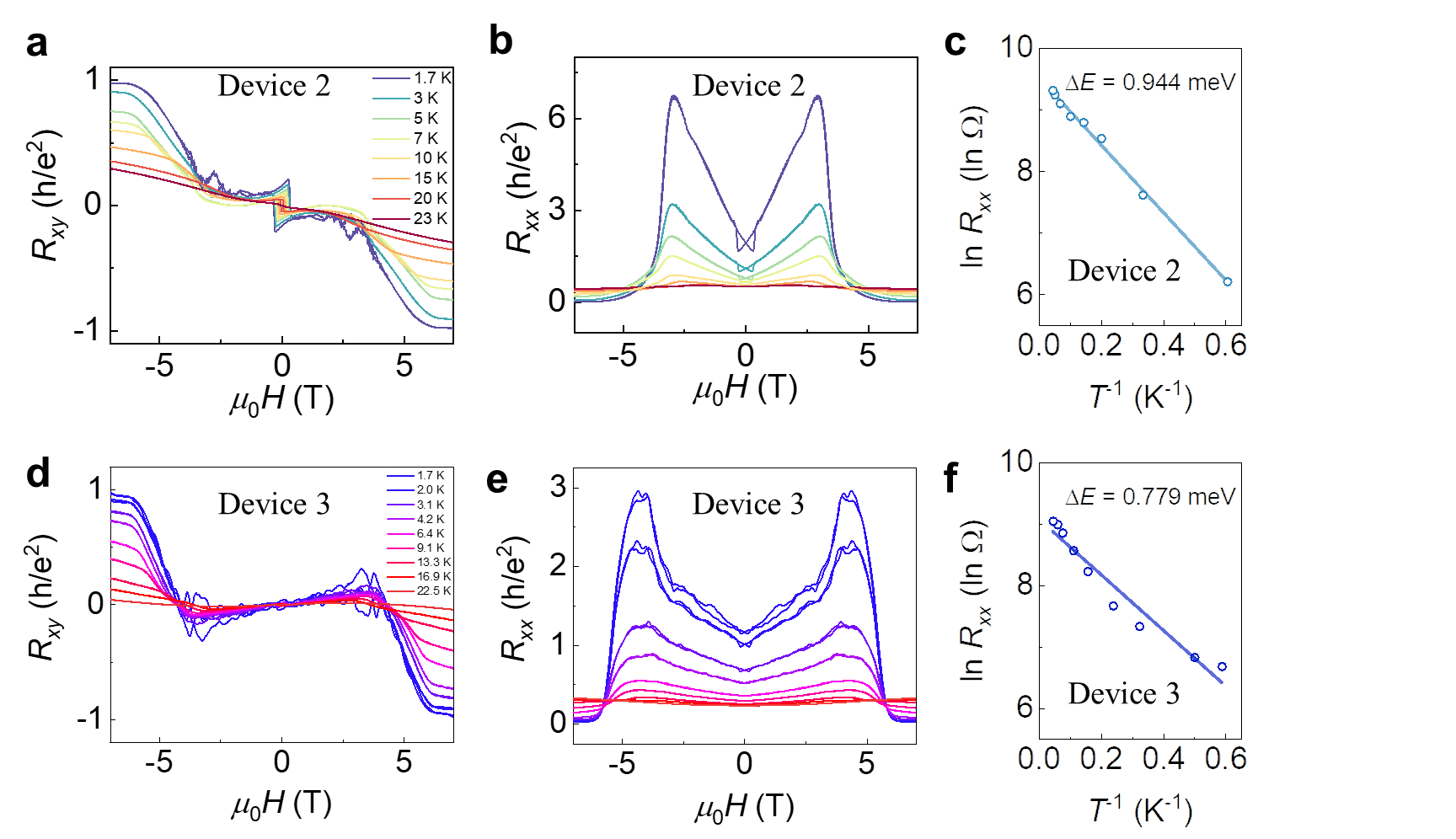 |
| --- |
| **Supplementary Fig. 4: Temperature dependence of Hall resistance and longitudinal resistance as a function of the magnetic field.** **a**, **b** and **c** The temperature dependent Hall resistance and longitudinal resistance of the 5-SL MBT (Device 2) and the estimated activation energy gap of 0.944 meV. The measurement is performed with the bottom gate voltage around the CNP of $V_{g}^{CNP}$=22 V. **d**, **e** and **f** The temperature dependent Hall resistance and longitudinal resistance of the 4-SL MBT (Device 3) and the estimated activation energy gap of 0.779 meV. The measurement is performed with the bottom gate voltage around the CNP of $V_{g}^{CNP}$=13 V. |

**Supplementary Note 3: Raw data and antisymmetrized data of Device 1**

| 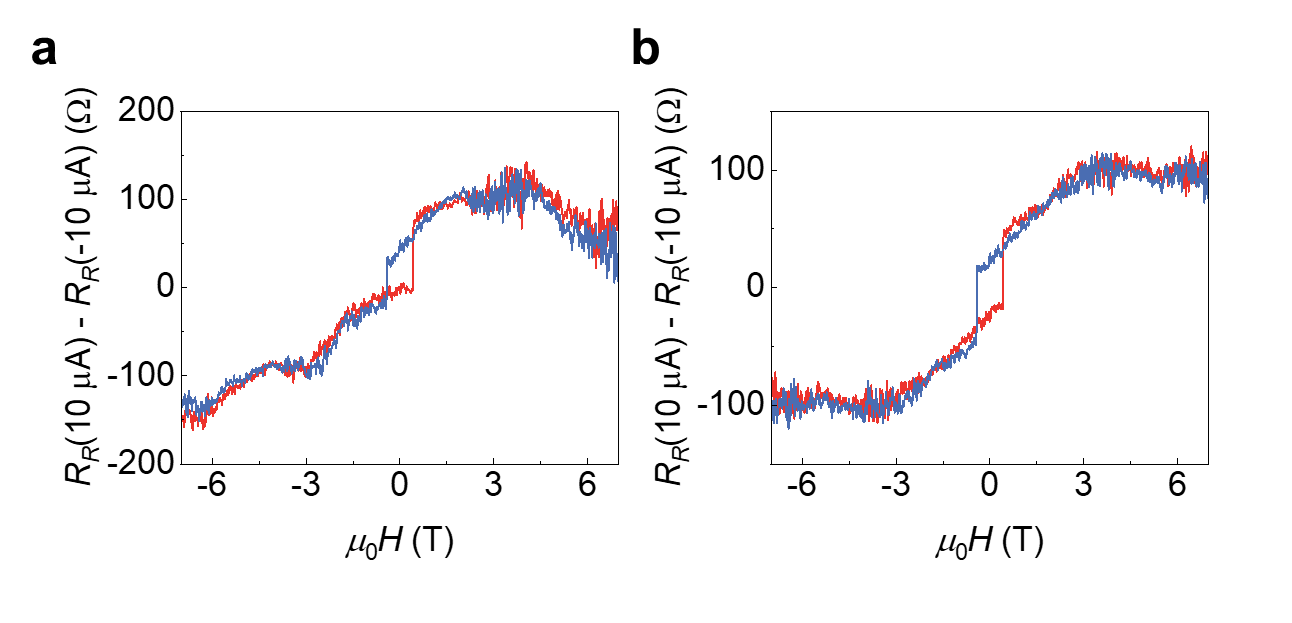 |
| --- |
| **Supplementary Fig. 5: Raw data and antisymmetrized data measured with DC current of Device 1.** The measurement temperature is 11 K, with a top gate voltage of -2 V. **a**, Raw data of the magnetic field dependence of the resistance difference between $R_{R}\left( 10 \mu A \right)$ and $R_{R}\left( -10 \mu A \right)$ . **b**, Antisymmetrized data of the magnetic field dependence of the resistance difference between $R_{R}\left( 10 \mu A \right)$ and $R_{R}\left( -10 \mu A \right)$. |

**Supplementary Note 4: Non-reciprocal charge transport in Device 2**

We have shown the gate dependent non-reciprocal charge transport in a 5-SL MBT (Device 1) in Fig. 4 of the main text. Here we show the gate dependent non-reciprocal resistance and Hall resistance in another 5-SL MBT (Device 2). The AC current $I^{RMS}$ injected into the sample is 1 $\mu A$.

| 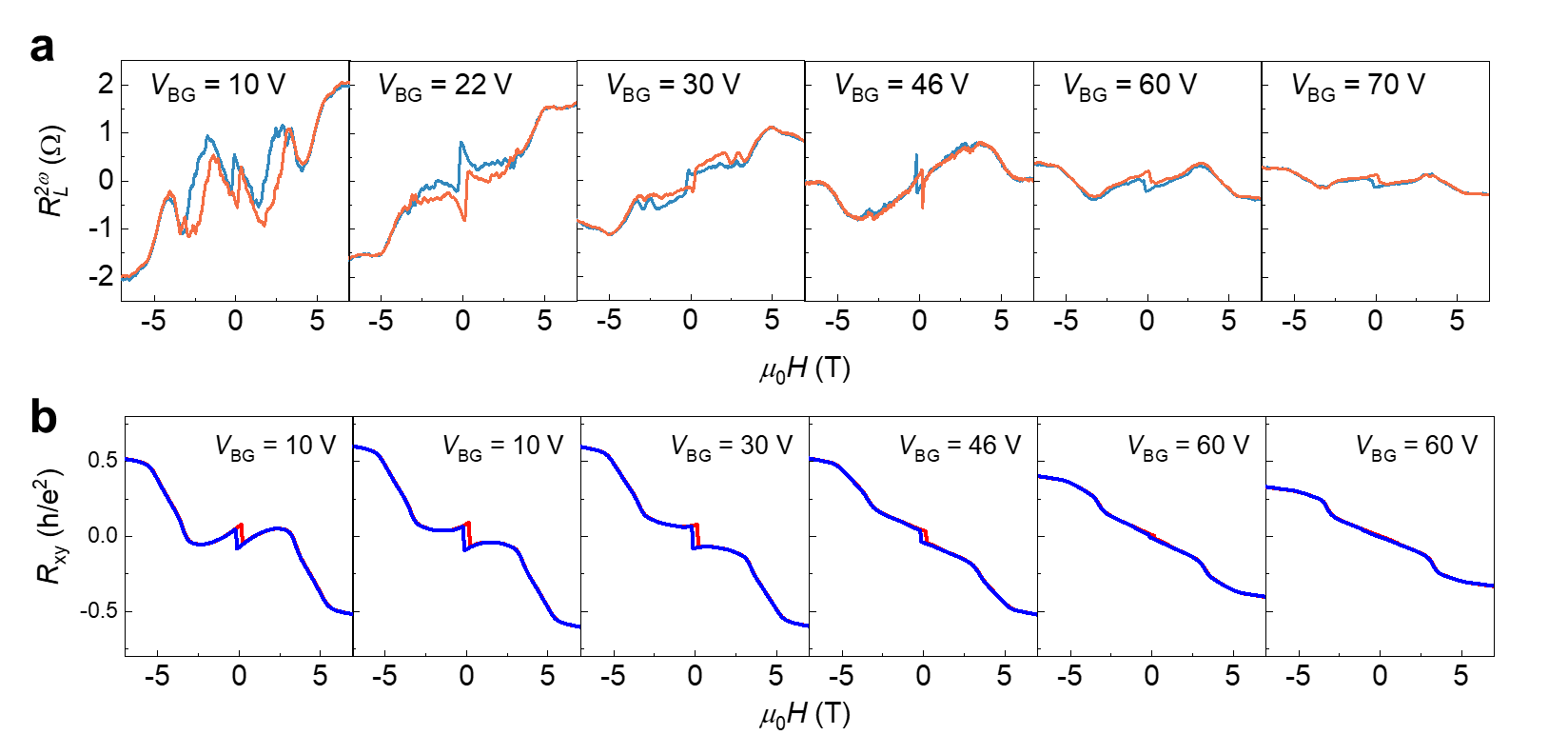 |
| --- |
| **Supplementary Fig. 6: Non-reciprocal charge transport and Hall resistance of Device 1 (5-SL) at different back gate voltage.** Two electrodes at the left sides of the sample are used to measure the non-reciprocal resistance. The measurement is performed at 10 K with AC current $I^{RMS}=1 \mu A$. **a**, Non-reciprocal resistance. **b**, Hall resistance. |

We also summarize the gate dependent non-reciprocal resistance under -7 T out-of-plane magnetic field in Supplementary Fig. 7. Under -7 T out-of-plane magnetic field, at the CNP with bottom gate voltage around $V_{\mathrm{BG}}^{\mathrm{CNP}}$ = 22 V, the Hall resistance reaches the maximum and the longitudinal resistance reaches the minimum as shown in Supplementary Fig. 7a. In Supplementary Fig. 7b, we show the non-reciprocal resistance measured at the left edge of the Device 2.

| 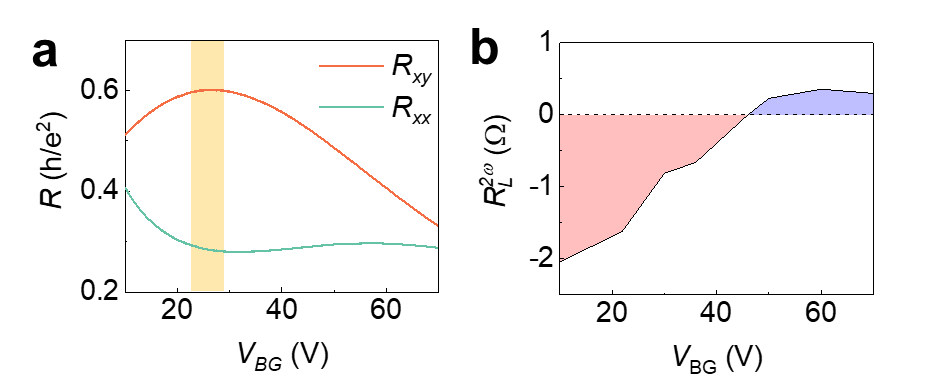 |
| --- |
| **Supplementary Fig. 7: The gate dependent non-reciprocal transport in 5-SL MBT (Device 2). a** Hall resistance and longitudinal resistance as a function of the bottom gate voltage. The measurement is performed at 10 K under -7 T magnetic field. The Hall resistance reached the maximum and the longitudinal resistance reaches the minimum at the CNP. **b** The non-reciprocal resistance at the left edge as a function of the bottom gate voltage. |

**Supplementary Note 5: Discussion about the thermoelectric contribution**

The thermal gradient $\nabla T$ that develops in the sample will result in a voltage perpendicular to both the thermal gradient and the magnetization direction. However, the second harmonic resistance resulting from the Nernst effect reverses the sign when the Fermi level is across the charge neutrality. In contrast to Nernst effect, our data show a large second-harmonic resistance at the charge neutrality.

We also carefully estimated the non-reciprocal resistance originating from Nernst effect. The reported Nernst coefficient of MnBi_2_Te_4_ under 9 T out-of-plane magnetic field is less than 1 $\mu$V/K^3^. At 10 K, when the injected current is 10 $\mu$A, the clear hysteresis loops indicate that the sample temperature is below its N$\acute{e}$el temperature 23 K. This means temperature increase due to the Joule heating is smaller than 13 K. The maximum resistance difference between *R*(-10 $\mu$A) and *R*(10 $\mu$A) resulting from Nernst effect should be 2.6$\Omega$. The experimental observation as shown in Fig. 2b in the main text is 100 $\Omega$, which is much larger than that we could expect from Nernst effect. Therefore, the non-reciprocal resistance observed in MnBi_2_Te_4_ cannot be attributed to the thermoelectrical effect.

**Supplementary Note 6: Current magnitude dependent non-reciprocal resistance**

We measured non-reciprocal resistances at various current magnitude. We show the data in Supplementary Fig. 8. The non-reciprocal resistance scales linearly with the injected AC current. The linear response is consistent with our phenomenological model (Eq1).

| 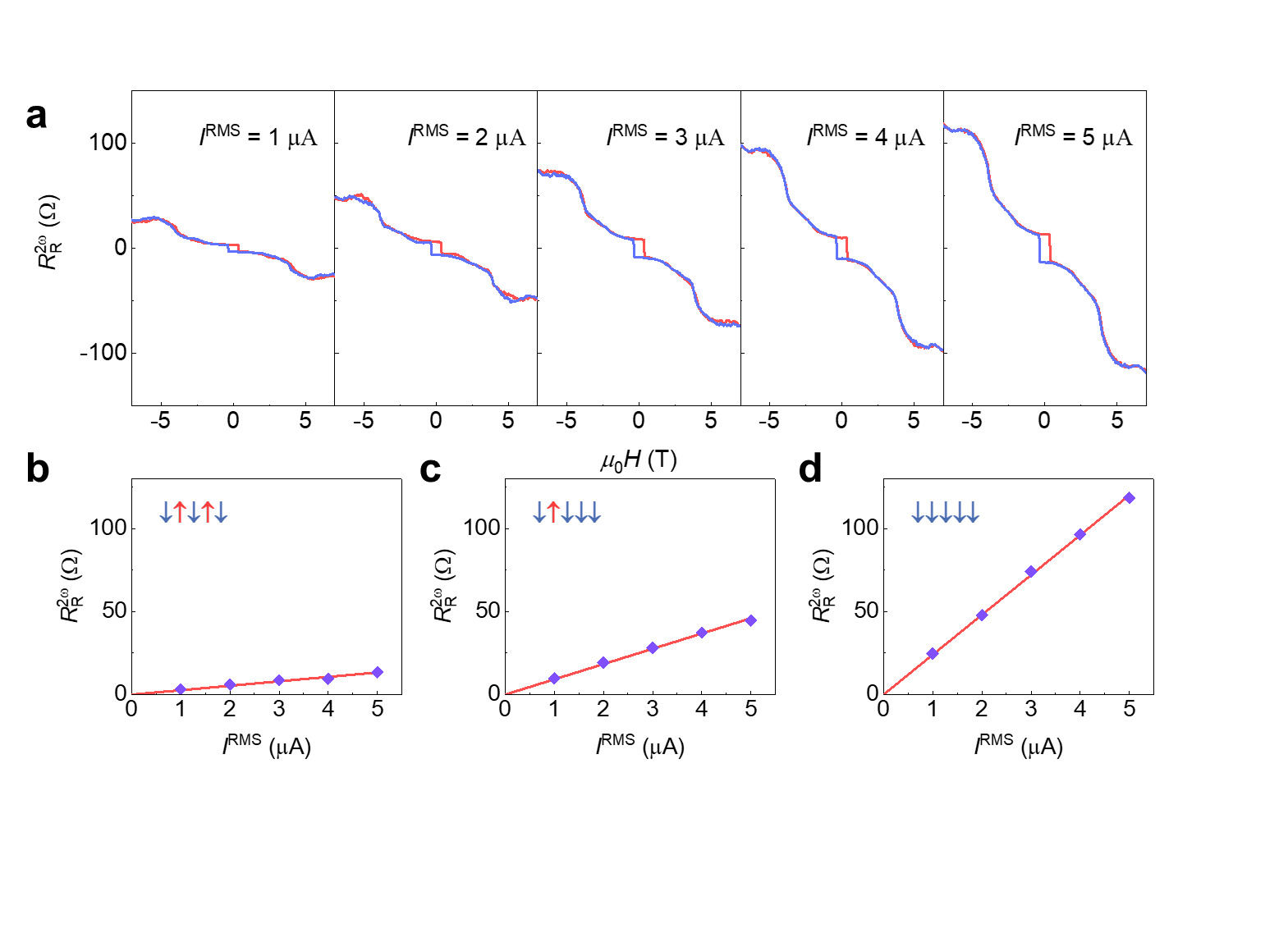 |
| --- |
| **Supplementary Fig. 8:** **Current magnitude dependent non-reciprocal resistance of S4.** All measurements are performed at 10 K. The sample is slightly hole doped. **a**, Out-of-plane magnetic field dependent non-reciprocal resistance with various current. All data are antisymmetrized. **b**, **c** and **d**, Current magnitude dependent non-reciprocal resistance at the magnetic state $\downarrow\uparrow\downarrow\uparrow\downarrow$, $\downarrow\uparrow\downarrow\downarrow\downarrow$, and $\downarrow\downarrow\downarrow\downarrow\downarrow$. |

**Supplementary Note 7: Vanished non-reciprocal charge transport at high temperature**

We show the effect of temperature on the non-reciprocal resistance in Supplementary Fig. 9. In Device 3, the non-reciprocal resistance vanishes when the temperature is 30 K, which is higher than its N$\acute{e}$el temperature. The vanished non-reciprocal resistance corresponds to magnetization $M\approx0$. Under this condition, the current relevant term in Equation 1 of the main text vanishes.

| 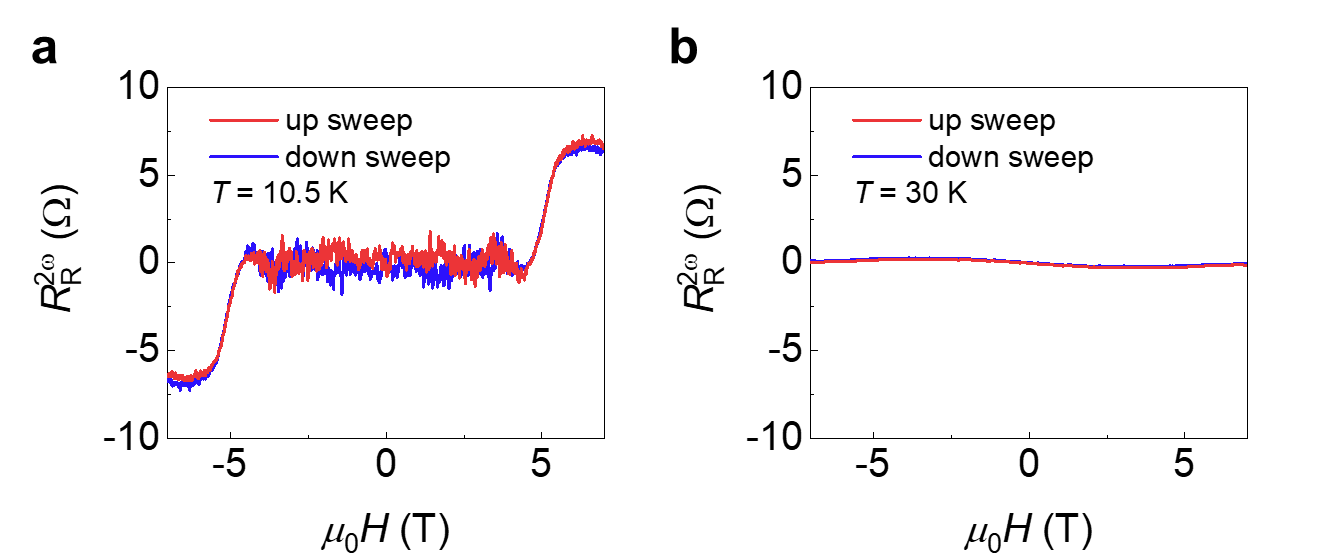 |
| --- |
| **Supplementary Fig. 9: The non-reciprocal resistance of 4-SL MBT device at different temperatures. a** and **b**, The non-reciprocal resistance in Device 3 (4-SL) measured at 10.5K and 30 K, respectively. The measurements are performed with top gate voltage of 3 V. |

**Supplementary Note 8: Comparison of the non-reciprocal resistance in 5-SL (S1) and 4-SL (S3) MnBi_2_Te_4_**

To compare the non-reciprocity in two samples, we calculate the magnitude of $\gamma.$ The $\gamma$ is calculated by $\gamma=\sqrt{2}R_{xx}^{2\omega}/(R_{0}\cdot I^{RMS})$, where $R_{xx}^{2\omega}$ is defined as $R_{xx}^{2\omega}=V_{xx}^{2\omega}/I^{RMS}$. To clearly show how we obtain the value of $\gamma$, we show the out-of-plane magnetic field dependent longitudinal resistance and non-reciprocal resistance in Supplementary Fig. 10.

| 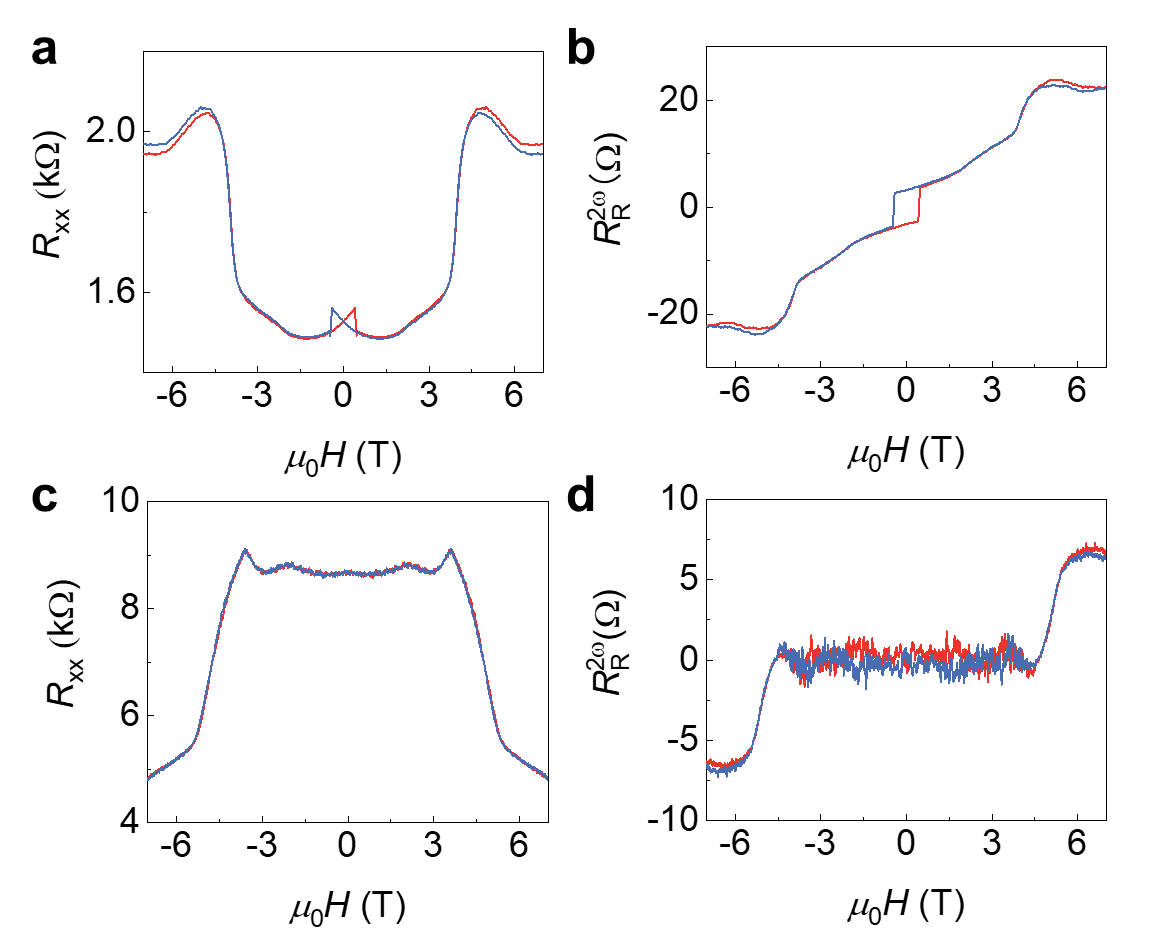 |
| --- |
| **Supplementary Fig. 10: Comparison of the non-reciprocal resistance in 5-SL (S1) and 4-SL (S3) MnBi_2_Te_4_.** For the 5-SL MnBi_2_Te_4_, the applied current $I^{RMS}$ is 5 $\mu$A with a top gate voltage of 3 V. For the 4-SL MnBi_2_Te_4_, the applied current is 5 $\mu$A with a top gate voltage of 3V. **a**, Out-of-plane magnetic field dependent longitudinal resistance of the 5-SL MnBi_2_Te_4_ (Devise 1). **b**, Out-of-plane magnetic field dependent non-reciprocal resistance of the 5-SL MnBi_2_Te_4_ (Device 1). **c**, Out-of-plane magnetic field dependent longitudinal resistance of the 4-SL MnBi_2_Te_4_(Device 3). **d**, Out-of-plane magnetic field dependent non-reciprocal resistance of the 4-SL MnBi_2_Te_4_ (Device 3). |

**Supplementary Note 9: Magnetic states of 5-SL at different gate voltage**

We study the effect of gate voltage on magnetic states in 5-SL MBT. Supplementary Fig. 11 shows field dependent Hall resistance at top gate voltage of -5 V, 0 V and 4 V of the 5-SL MBT (Device 1). We note the magnitude of the Hall resistance changes at different gate voltage, but the sign does not change. This indicates the magnetic states of 5-SL MBT under zero magnetic field are independent to the gate voltage in our device.

| 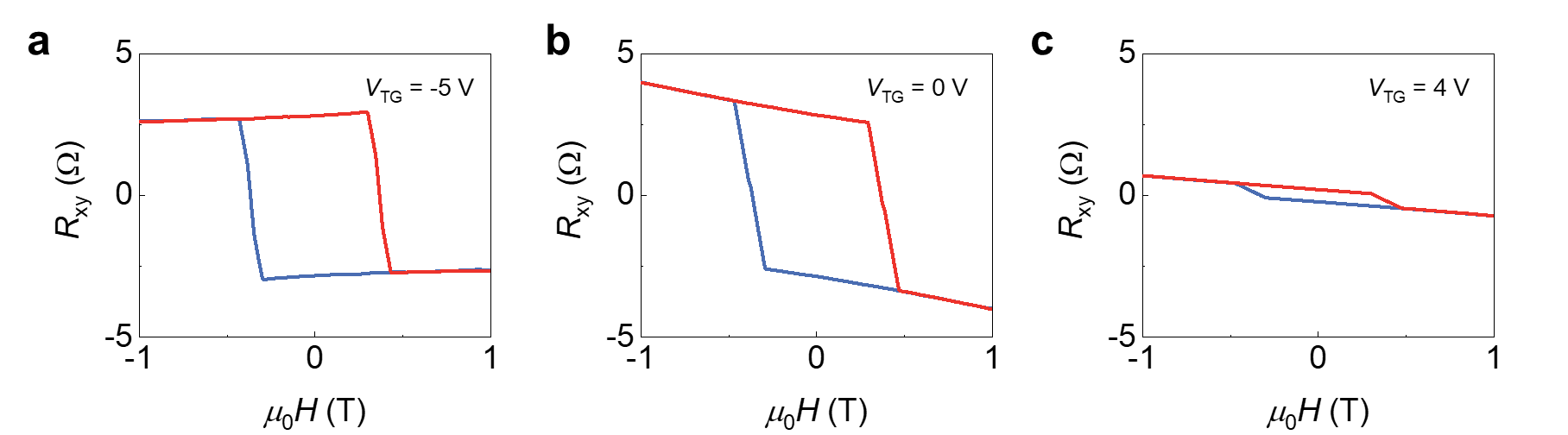 |
| --- |
| **Supplementary Fig. 11: The Hall resistance of 5-SL MBT device(Device 1) at different gate voltage.** Field dependent Hall resistance at top gate voltage of -5 V, 0 V and 4 V of the 5-SL MBT (Device 2). |

In a magnetic topological insulator, the anomalous Hall resistance originates from the combined magnetization and topological band structure. By measuring Hall resistance, we are unable to confirm whether the magnetization is changed upon applying gate voltage. To confirm the effect of gate voltage on magnetization, we perform optical magnetic circular dichroism (MCD) measurements for a 5-SL MnBi_2_Te_4_ (Device 5) at different gate voltages. As shown in Supplementary Fig. 12a, the charge neutrality point is at V*_t_* = -9.3 V, indicated by the resistance peak. We perform optical MCD measurements using the setup shown in Supplementary Fig. 12b. We show MCD loops at top gate voltage of -14 V, 9.3 V and 0 V in Supplementary Fig. 12c. The magnetic field is swept from -1 T to 1 T. Since the magnitude of MCD is proportional to the magnetization, unchanged MCD loops at different gate voltages reveal that the magnetization is gate independent in this field regime.

Based on the above discussions, the shrinking non-reciprocal resistance hysteresis loops at some gate voltages originate from the doping level change, during which the magnetization is unchanged.

| 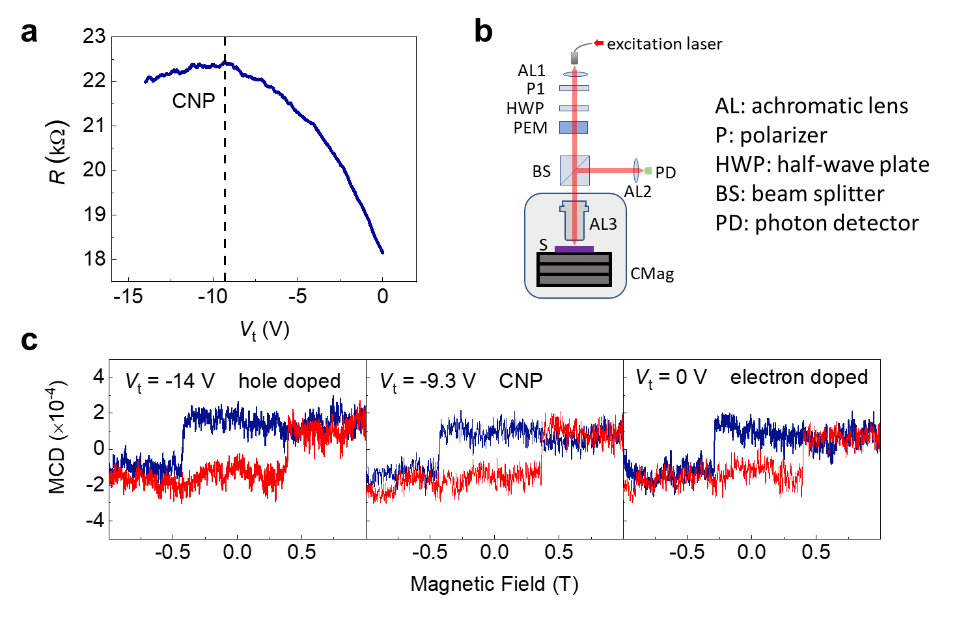 |
| --- |
| **Supplementary Fig. 12: Gate voltage independent magnetization.** **a**, Gate voltage dependent resistance of 5-SL MnBi_2_Te_4_ under zero magnetic field. The resistance shows a peak at the charge neutrality point. **b**, Optical MCD setup. The wavelength of the excitation laser is a 632.8 nm and the laser power is around 1 $\mu$W. **c**, Optical MCD at gate voltage of -14 V, -9.3V and 0 V. |

**Supplementary Note 10: Magnetic field angle dependent non-reciprocal charge transport**

We performed the magnetic-field-direction dependent measurements for a 5-SL MnBi_2_Te_4_ (Device 4). Supplementary Fig. 13a shows the schematic structure of the MnBi_2_Te_4_ device. For magnetic-field-direction dependent measurements, the 7 T magnetic field is rotated in the x-z plane. We first measure the Hall resistance under the out-of-plane magnetic field at *V*_b_ = 0 V and *V*_b_ = -20 V, respectively. The results are shown in Supplementary Fig. 13b. The sample is hole doped at *V*_b_ = 0 V, and applying a negative bottom gate voltage increase the hole density in the sample. Then we measure the Hall resistance and non-reciprocal resistance when rotating the magnetic field. We show the results in Supplementary Fig. 13c to f. At *V*_b_ = 0 V, we observe a dip of $R_{R}^{2\omega}$ when the $R_{xy}$reaches the maximum value under an out-of-plane magnetic field. However, this feature disappears at *V*_b_ = -20 V, where $R_{R}^{2\omega}$ increases monotonically as $R_{xy}$ increasing. This behavior agrees with previous reported non-reciprocal charge transport in Cr-doped (Bi,Sb)_2_Te_3_ and confirms that the non-reciprocal resistance in MnBi_2_Te_4_ originates from the interplay between chiral edge states and bulk states.

| 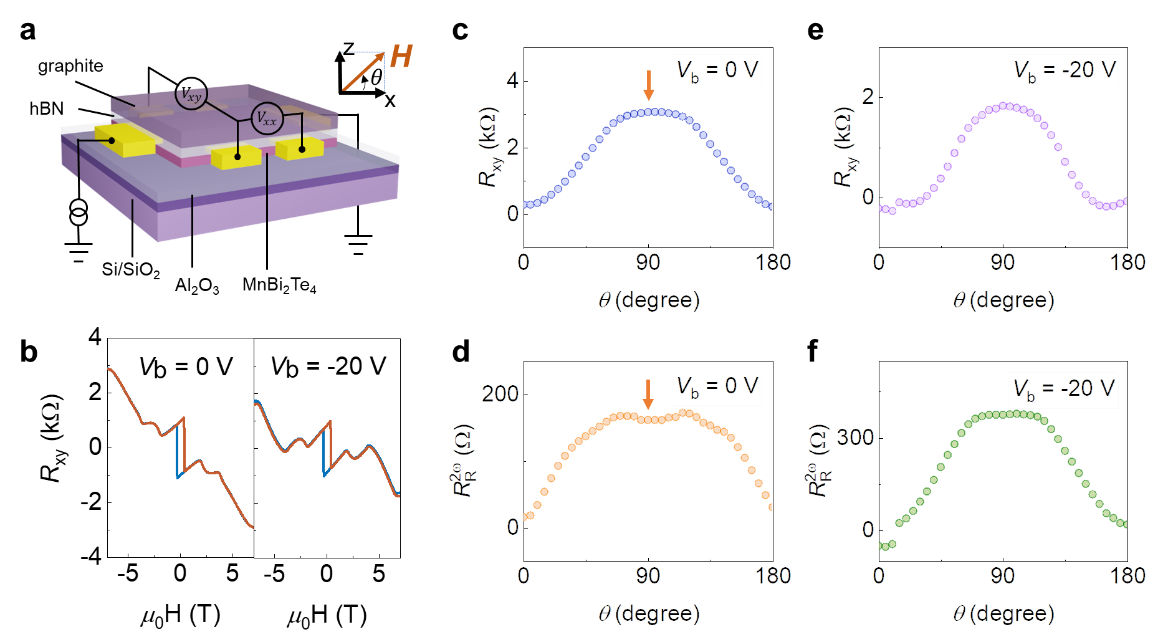 |
| --- |
| **Supplementary Fig. 13:** **Magnetic-field-direction dependent measurements of a 5-SL MnBi_2_Te_4_ (Device 4).** All measurements are performed at 10 K with $I^{RMS}=$3 $\mu A$. **a**, The schematic illustration of magnetic-field-direction dependent measurements. **b**, Hall resistance at *V*_b_ = 0 V and *V*_b_ = -20 V. **c** and **d**, magnetic-field-direction dependent Hall resistance and non-reciprocal resistance at *V*_b_ = 0 V under -7 T. **e** and **f**, magnetic-field-direction dependent Hall resistance and non-reciprocal resistance *V*_b_ = -20 V under -7 T. |

Non-reciprocal charge transport is enabled in quantum anomalous Hall systems, originating from the interplay between chiral edge states and bulk states. In this sense, non-reciprocal charge transports in MnBi_2_Te_4_ and Cr-doped (Bi,Sb)_2_Te_3_ are similar. However, MnBi_2_Te_4_ shows some unique features, which is different from that in Cr-doped (Bi,Sb)_2_Te_3_. We summarized the difference of non-reciprocal charge transports between MnBi_2_Te_4_ and Cr-doped (Bi,Sb)_2_Te_3_:

1. Multiple non-reciprocal resistance states originating from the antiferromagnetic interlayer coupling. MnBi_2_Te_4_ is an A-type antiferromagnet, where magnetic moments are ferromagnetically coupled within the SL and antiferromagnetically coupled between two neighboring SLs. For a 5-SL MnBi_2_Te_4_, applying an out-of-plane magnetic field results in various magnetic states, such as $\uparrow\downarrow\uparrow\downarrow\uparrow$, $\downarrow\uparrow\downarrow\uparrow\downarrow$, $\uparrow\uparrow\uparrow\downarrow\uparrow$, $\downarrow\downarrow\downarrow\uparrow\downarrow$, $\uparrow\uparrow\uparrow\uparrow\uparrow$, and $\downarrow\downarrow\downarrow\downarrow\downarrow$. Tuning the magnetization of the sample effectively modifies bulk-to-edge conduction ratio, manifesting in the Hall resistance plateaus and non-reciprocal plateaus.
2. Layer-number manipulation of the non-reciprocal charge transport. The antiferromagnetic interlayer coupling makes even-SL and odd-SL MnBi_2_Te_4_ magnetically different under zero magnetic field. Without external magnetic field, odd-SL-number MnBi_2_Te_4_ is an uncompensated antiferromagnet with nonzero net magnetic moments, and even-SL-number MnBi_2_Te_4_ is a fully compensated antiferromagnet with zero net magnetic moments. Because of the different magnetic states, 5-SL samples show nonzero non-reciprocal resistance but 4-SL samples show almost vanished non-reciprocal resistance without magnetic field.

References:

1 Deng, Y. *et al.* Gate-tunable room-temperature ferromagnetism in two-dimensional Fe_3_GeTe_2_. *Nature* **563**, 94-99 (2018).

2 Deng, Y. *et al.* Quantum anomalous Hall effect in intrinsic magnetic topological insulator MnBi_2_Te_4_. *Science* **367**, 895-900 (2020).

3 Zhang, H., Xu, C., Lee, S., Mao, Z. & Ke, X. Thermal and thermoelectric properties of an antiferromagnetic topological insulator MnBi_2_Te_4_. *Physical Review B* **105**, 184411 (2022).
